# Skin Sensitization Testing Using New Approach Methodologies

**DOI:** 10.3390/toxics13050326

**Published:** 2025-04-22

**Authors:** Judy Strickland

**Affiliations:** Inotiv, Inc., Morrisville, NC 27560, USA; judystrickland@frontier.com

Chemical regulatory authorities require skin sensitization information for a number of chemical sectors, such as cosmetics, pesticides, newly marketed chemicals, household products, and topical pharmaceuticals [1]. If regulatory agencies need such information, then chemical and product developers and distributors also need it. Usually, the information needed is skin sensitization hazard, a binary classification to denote whether the substance has the potential to induce skin sensitization or not, and, if the substance has skin sensitization potential, skin sensitization potency is needed. Potency information is provided as a multilevel categorical classification or a point estimate, sometimes referred to as a “point of departure” (POD), which is a toxicity value used for risk assessment. Such information is typically determined in animal tests. However, skin sensitization is one of the regulatory toxicity endpoints seeing much progress in the uptake of new approach methodologies, which are non-animal skin sensitization evaluations, for regulatory and developmental purposes. The mechanisms of skin sensitization initiated by binding to skin proteins is well-characterized by an adverse outcome pathway published by the Organization for Economic Cooperation and Development (OECD) [2]. Test method developers have used the key events in the adverse outcome pathway as the targets for developing non-animal tests that assess whether a chemical or product may activate them.

This Special Issue, “Skin Sensitization Testing Using New Approach Methodologies”, shows that there is much research and application activity in this area, as evidenced by the 10 articles contributed.

These articles provide a broad coverage of the area of new approach methodologies for skin sensitization assessments. A number of articles involve test method development. Fritch et al., Contribution 10, documents the problem formulation stage of test method development for T-cell activation, which is key event 4 of the adverse outcome pathway for skin sensitization produced by chemicals that bind to skin proteins [2]. Although there aresome non-animal tests that measure T-cell activation to assess a substance’s potential for skin sensitization [3,4], there are no regulatory tests established in harmonized international test guidelines. Contribution 10 shows that known approaches to assess T-cell activation are challenged by chemical-mediated toxicity, inefficient or unknown generation of T-cell epitopes, and low throughput. Contribution 10 reviews the available solutions and strategies to confirm in vitro T-cell signals and concludes that application and standardization are necessary to define chemical applicability domains and to strengthen the role of T-cell tests in regulatory risk assessment.

In Contribution 1, Burla et al. report work to further the development of a non-animal test for respiratory sensitizers, which are classified as substances of very high concern under the European Commission Regulation for the Registration, Evaluation, Authorization and restriction of Chemicals (REACH). There are currently no animal or non-animal test methods approved for regulatory use in evaluating chemicals for respiratory sensitization hazards. Respiratory sensitizers test positive in the existing animal skin sensitization tests, but these tests do not provide enough information to distinguish between skin and respiratory sensitizers. ALIsens^®^, a three-dimensional in vitro alveolar model, had previously been evaluated for the classification of respiratory sensitizers [5]. Contribution 1 continues the evaluation of ALIsens^®^ by establishing its efficacy in discriminating respiratory sensitizers from skin sensitizers and non-sensitizers.

Contributions 8, Ahmed et al., and 9, Martins-Ribeiro et al., describe a laboratory test for skin sensitization that has been adapted to detect the immunotoxicity of therapeutic biologics (Contribution 8), or antibodies damaged by heat, which produces antibody aggregation (Contribution 9). These tests are very human-relevant. Both investigations expose human skin explants to evaluate histopathology, and also expose peripheral blood mononuclear cells from the same donor to evaluate T-cell proliferation or cytokine levels. Contributions 8 and 9 show that the adapted skin sensitization tests [3,4] were applicable to identifying adverse immune reactions in addition to skin sensitization.

A number of contributions to this Special Issue build and evaluate computational models to classify test substances for skin sensitization hazard or to place sensitizers in potency categories. In Contribution 2, Tieghi et al. develop and evaluate an in silico model that predicts human skin sensitization hazard and potency categories per the Globally Harmonized System of Classification and Labelling of Chemicals [6]. Human skin sensitization data were used to build this model and the model is freely available. Such a model can be easily implemented in the early stages of drug or chemical development and testing to minimize resource waste and facilitate the early stages of molecule development while reducing animal testing. Thus, these models are useful even if they do not progress towards regulatory acceptance.

Mohoric et al. (Contribution 3) and Tourneix et al. (Contribution 6) report on Bayesian models that combine data streams from in vitro and in silico results to estimate substance potency for inducing skin sensitization. Both models were based on the work of Jaworska et al. [7,8]. Animal data from the murine local lymph node assay (LLNA) were also used to build these models. Both contributions build and evaluate Bayesian models that predict LLNA potency in four categories as well as PODs for sensitizers that can be used for risk assessment. However, the models reported in Contributions 3 and 6 use different potency categories and different methods for predicting the LLNA potency value. The models from both contributions provide confidence indications for each prediction. Contributions 3 and 6 show that risk assessment can be performed without animal data.

Gradin et al. does as well, in Contribution 4, and provides a much simpler and less data intensive approach to developing a POD for skin sensitization risk assessment. The approach uses the information from only one in vitro test, which is based on gene expression, the GARDskin Dose–Response assay. The best performing model, which estimates PODs, is a regression of no-expected-sensitization-induction levels based on human and LLNA data (in vivo PODs) on the assay readout using only an intercept parameter.

In Contribution 5, Wareing et al. provides an in vitro test method evaluation. It evaluates the applicability of three key event assays included in the existing OECD test guideline methods [9,10,11], one for each of the first three key events, for testing inorganic nanomaterials for skin sensitization potential. Although the in vitro methods were modified in an attempt to adjust their suitability to nanomaterials, they were originally developed and evaluated for small organic molecules and were not a good fit for testing inorganic nanomaterials.

One contribution, 7, by Juncan et al., provides a case study showing how new approach methodologies for skin sensitization can be applied to toxicity evaluation during the product development of cosmetic ingredients. Skin sensitization was one of the toxicity endpoints assessed. Evaluations of stability, physicochemical properties, and microbiology were also performed. Contribution 7 reports on the development and evaluation processes all the way from the assessment of several candidate ingredients to be included in the new formulation to assessments of the final formulation. Contribution 7 shows how a novel proprietary in silico model that predicts skin sensitization can be used to evaluate ingredients during product development. The in silico evaluation was verified with clinical testing.

The contributions to this Special Issue, “Skin Sensitization Testing Using New Approach Methodologies”, highlight a number of data gaps. Skin sensitization test results or model predictions are typically not considered alone to make a decision on how to classify a substance. Usually, all information about the substance in question is considered, including, but not limited to, physicochemical properties, results for other toxic endpoints, and the known skin sensitization potential of similar substances. A decision tree on how to apply the results of new approach methodologies for skin sensitization hazard and potency is needed in order to integrate the skin sensitization information with other pertinent chemical information. This especially applies to the in silico methods described in Contribution 2 and the individual in vitro test methods described in Contributions 4 and 5. However, the Bayesian network models described in Contributions 3 and 6 already use a number of different data streams. Would these model predictions be sufficient as stand-alone hazard or potency classifications? More research and evaluation is needed to identify the best approach for the application of these methods.

Contribution 7 reminds us that the existing OECD test guidelines are not applicable to all types of substances. The existing guidelines are intended to target mechanisms relevant to organic substances that induce skin sensitization by binding to skin proteins. Inorganic nanomaterials (as is the case in Contribution 7), metals such as nickel, and biological agents may induce skin sensitization by other mechanisms [2]. Thus, research and development of applicable test methods to detect substances that induce sensitization by means other than binding to skin proteins is needed. Such test development and application of new approach methodologies for skin sensitization will continue to be a focus of the regulatory and regulated communities in order to reduce animal use for these assessments and provide faster and more human-relevant results.

## Data Availability

Not applicable.
